# Students’ perception of asynchronous versus synchronous distance learning during COVID-19 pandemic in a medical college, southwestern region of Saudi Arabia

**DOI:** 10.1186/s12909-023-04034-5

**Published:** 2023-01-23

**Authors:** Hassan A. Alzahrani, Ayed A. Shati, Mohammed A. Bawahab, Abdulaziz A. Alamri, Bahaeldin Hassan, Ayyub A. Patel, Mohammad Tauheed Ahmad, Walid Abd El Maksoud, Mohammed A. Alsaleem

**Affiliations:** 1 Department of Surgery, College of Medicine, King Khalid University, Abha, Saudi Arabia; 2 Department of Medical Education, College of Medicine, King Khalid University, Abha, Saudi Arabia; 3 Department of Child Health, College of Medicine, King Khalid University, Abha, Saudi Arabia; 4 Department of Obstetrics and Gynecology, College of Medicine, King Khalid University, Abha, Saudi Arabia; 5 Department of Biochemistry, College of Medicine, King Khalid University, Abha, Saudi Arabia; 6 Department of Family and Community Medicine, College of Medicine, King Khalid University Abha, Abha, Saudi Arabia

**Keywords:** Online learning, Time management, Interactive participation, COVID-19, Learning, Synchronous, Asynchronous

## Abstract

**Background:**

COVID-19 preventive measures required a drastic shift to online teaching-learning in most of countries. Institutions used different combinations of live online lectures (synchronous) requiring students to attend the class in real-time, as well as recorded lectures uploaded by the instructors to be accessed by students as per their own convenience (asynchronous). We undertook this study to assess and compare the perceptions of students regarding their learning experiences in the synchronous versus asynchronous mode of instruction using  their teaching-learning during the compulsory online mode of instruction at the peak of the COVID-19 pandemic.

**Methodology:**

This cross-sectional questionnaire-based study received responses from 122 final-year medical students studying at the College of Medicine, King Khalid University, Abha, Saudi Arabia. An online 5-point Likert scale-based questionnaire was used to collect data regarding experience and perception towards synchronous and asynchronous learning. Statistical analysis was done using Statistical Package for Social Sciences (SPSS) version 21.0. A *P*-value less than 0.05 was considered significant.

**Result:**

All the students found both synchronous and asynchronous learning to be equally satisfying, enjoyable and comfortable. No statistically significant difference was found when both the methods were analyzed for enhancement of knowledge. The students opined that asynchronous learning helped them manage their time better whereas synchronous learning encouraged more interaction during the live lectures.

**Conclusion:**

Overall, the students' perceptions regarding both synchronous and asynchronous online learning were positive. As both methods have their advantages/limitations, a mix of both synchronous and asynchronous methods may be adopted depending upon the content of the topic and the desired learning outcomes.

## Introduction

The learning environment is that which surrounds learning and it can be in a face-to-face format and/or a virtual format. Most comprehensively, it has been defined as “the social interactions, organizational culture and structures, and physical and virtual spaces that surround and shape the learners’ experiences, perceptions, and learning” [1]. The COVID-19 pandemic affected all aspects of life all over the world including the Kingdom of Saudi Arabia. The lockdown resulted in the closure of schools, colleges, and universities. The rapid shift to the virtual space of the learning environment was the solution [2]. In the virtual space, two different forms are adopted. First, synchronous form which is defined “as live streaming video and/or audio with instantaneous feedback” [3]. This form allows simultaneous gathering and reaction between teachers and students. Second, asynchronous form, which is defined “as communication occurring through the use of email and discussion boards, with the instructor playing a larger role as facilitator between students” [4]. This form requires teachers to prepare course material in advance to be accessed by students without real-time interaction.

Each form has its advantages and disadvantages. For instance, live synchronous learning experiences have the advantage of immediate interactions and personal engagement. Moreover, synchronous learning prevents misunderstanding of the subject matter. On the contrary, asynchronous learning experiences have the advantage of high temporal flexibility. The students can access their subject matters without facing the risk of technical challenges such as internet interruption [5, 6].

The implementation of digital technology into health professions education is becoming imperative in the current digital era. More opportunities for learning by enhancing competency-based learning rather than time-based learning is currently available [7–9]. Medical education, which has been traditionally done in a face-to-face format has incorporated elements of virtual learning through the emerging models of pedagogy. Student use of the learning management system has been adopted in most of the countries and where resources permit their implementation. It is not uncommon to see certain courses in undergraduate medicine programs in high resource institutions, delivered exclusively online as per suitability. Apart from that online self-directed learning through the learning management system has been applied to varying degrees as a significant component of the teaching-learning in methods of instruction such as flipped classroom, problem-based learning, team-based learning etc. [10–12] Delivery of instruction through the synchronous and asynchronous modes have their innate advantages and limitations. It is also important to gauge the students’ perceptions regarding the two modes. There is a dearth of studies internationally studying this specific issue in the context of medical education. Hence, the compulsory shift to online learning during the COVID-19 inspired us to undertake this research to study the perceptions of students regarding synchronous versus asynchronous mode of online instruction delivery.

The main goal of our study is to determine medical students’ satisfaction with online learning experiences. Students’ perceptions of synchronous versus asynchronous mode of instruction during the COVID-19 pandemic were measured and compared.

## Methods

### Study design and setting

A cross-sectional study was conducted in the College of Medicine, King Khalid University, Abha, Saudi Arabia.

### Study population

The participants were the final year, (level 12) medical students of the surgery course. Learning experiences were delivered to students using the two online methods. The first method was the synchronous form (live interactive sessions using a cloud-based video conferencing service). The second method was the asynchronous form (voice record PowerPoint presentations and discussion forums that were made available via a learning management system (LMS) “Blackboard”, a web-based learning environment).

### Data collection

A questionnaire comprising of 12 items to elicit the responses of the students about their perceptions regarding enjoyment of the teaching method, enhancement in knowledge, time-management, interactiveness, ease of learning as well as overall satisfaction with the synchronous vs asynchronous modes, was developed. Apart from these variables, there were also questions regarding demographic details such as age and gender of the participants. Answers were based on a 5-point Likert scale. Strongly agree was represented by 5, strongly disagree was represented by 1, and 3 represented a neutral response. The questionnaire was pilot tested and necessary modifications were made. After validation (Cronbach α = 0.85), the finalized questionnaire was structured in the Google forms format for online dissemination. After the completion of the respective courses, the final year medical students were sent the electronic forms with a request to participate in the survey. Out of the total 197 students surveyed, 122 students returned completed responses giving a response rate of 61.9%.

### Data analysis

The  data were collected, revised, coded, and entered into Statistical Package for the Social Sciences (SPSS) software program, version 21.0 (IBM Corp. Released 2013. IBM SPSS Statistics for Windows, Version 21.0. Armonk, NY: IBM Corp). Descriptive statistics including frequencies and percentages were used to describe the frequency of each categorical variable item. Chi-square and Fisher’s exact test were used to test for association between respondent characteristics and their selection preference. All statistical analyses were performed using two-tailed tests and α error of 0.05. A value of *P* ≤ 0.05 was considered to be statistically significant.

### Ethical approval

The study was approved by Ethics and Internal Review Board Committee (WCM# 2020–1401). Written consent was attached to each survey before response-questions.

## Results

A total of 122 final year medical students participated in the study, The mean age + standard deviation was (23.78 ± 2.76) years.

**Fig. 1:**
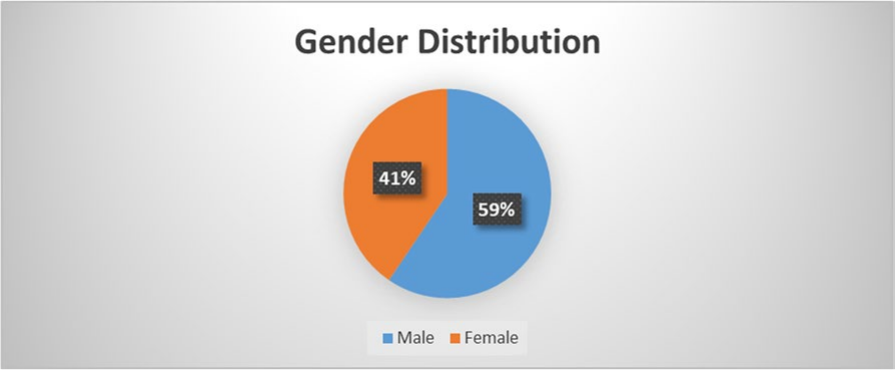
Gender distribution of the participants

Gender distribution among the study participants was 59% male students and 41% female students. (Fig. 1).

Internal consistency and split half reliability of the questionnaire items [12] were checked on 15% of the students’ sample and the Cronbach’s alpha score was 0.87 (Table 1).

**Table 1:** Item’s reliability statistics

Items	Cronbach’s Alpha if Item Deleted
1.1 I liked and enjoyed asynchronous (recorded) lectures	0.86
1.2 I liked and enjoyed synchronous (live) lectures	0.85
2.1 My knowledge was enhanced more with asynchronous (recorded) lectures	0.85
2.2 My knowledge was enhanced more with synchronous (live) lectures	0.85
3.1 My time was used better with asynchronous (recorded) lectures	0.85
3.2 My time was used better with synchronous (live) lectures	0.87
4.1 My level of participation was better with asynchronous (recorded) lectures	0.86
4.2 My level of participation was better with synchronous (live) lectures	0.86
5.1 My experience was easy and comfortable with asynchronous (recorded) lectures	0.85
5.2 My experience was easy and comfortable with synchronous (live) lectures	0.85
6.1 I was satisfied more with asynchronous (recorded) lectures	0.85
6.2 I was satisfied more with synchronous (live) lectures	0.86
Total items [12] standardization	0.87

We compared the student’s responses between synchronous and asynchronous learning (Table 2). No significant differences concerning the enjoyment of teaching methods, enhancement in knowledge, ease, and comfortable experiences and satisfaction were found between the two methods.

**Table 2:** Comparison of students' responses of synchronous and asynchronous learning

Items	Asynchronous		Synchronous		p- value
	Mean	S.D	Mean	S.D	
Enjoyment of teaching methods	3.14	1.43	3.27	1.26	0.60
Enhancement in knowledge	2.83	1.32	3.17	1.16	0.13
Better usage of time	3.71	1.38	2.71	1.30	*0.0001*
Level of interaction	2.60	1.35	3.10	1.17	*0.0320*
Ease and Comfortable experiences	3.20	1.44	3.11	1.19	0.71
Satisfaction	2.98	1.36	3.14	1.20	0.49

However, there were significant differences between asynchronous and synchronous learning regarding time management (3.10 ± 1.38 Vs 2.71 ± 1 30, *P* = 0.0001) and participant’s interaction (2.60 ± 1.35 Vs 3.10 ± 1.17, *P* = 0.0320) respectively. (Table 3).

**Table 3:** Comparison of students’ responses of synchronous and asynchronous learning by Gender

	Males					Females				
	Asynchronous		Synchronous		*p*-value	Asynchronous		Synchronous		*p*-value
Items	Mean	S.D.	Mean	S.D.		Mean	S.D.	Mean	S.D.	
Enjoyment of teaching method	3.00	2.14	3.2	2.16	0.56	3.17	4	3.35	2.22	0.77
Enhancement in knowledge	2.7	1.18	3.01	1.17	0.11	3.01	2.14	3.19	1.8	0.64
Better usage of time	3.57	2.14	2.9	1.45	0.02	3.88	1.98	2.49	1.25	0.001
Level of interaction	2.62	1.28	2.95	1.85	0.2	2.6	1.45	3.33	1.36	0.01
Ease and comfortable experiences	3.16	1.45	3.12	1.28	0.85	3.29	1.28	2.08	2.3	0.001
Satisfaction	2.89	1.55	3.06	1.45	0.48	3.05	1.27	3.15	1.98	0.76

We studied the differences between synchronous and asynchronous learning among male and female students (Table 3). We observed that both male and female students preferred asynchronous learning regarding time management (3.57 ± 2.14 Vs 2.90 ± 1.45, *P* = 0.0010), (3.88 ± 1.98 Vs 2.49 ± 1.25, *P* = 0.0010) respectively. (Table 3).

Female respondents reported significantly greater preference to participate in live sessions vs recorded lectures. (3.33 ± 1.36 Vs 2.60 ± 1.45, *P* = 0.0100). However, female students found asynchronous learning easy and comfortable as compared to synchronous learning (3.29 ± 1.28 Vs 2.08 ± 2. 30, *P* = 0.0010) (Table 3).

## Discussion

Since the WHO declared COVID-19 a pandemic, strict physical distancing regulations were put in place around the world to delay the spread of the pandemic. With this, almost all institutions came  to a standstill, including  educational institutions; with no sign of  slowing down of the pandemic and the emergence of new variants of the virus. Many medical schools turned to online mode of teaching. Although the online mode of teaching was followed in the medicine undergraduate programs in the Kingdom even before this pandemic, it was limited to assignment and group discussions [13]. The pandemic resulted in forced transformation from the conventional face to face teaching to the use of online mode of teaching which was new to many students. Hence, our study was done to evaluate the perspectives, experience, and preference with regards to online learning, both the synchronous form and the asynchronous form.

In our study, the students, both boys and girls, were comfortable with both synchronous and asynchronous methods of learning and found both methods equally enjoyable. This finding was further endorsed by the fact that they found both methods to be equally effective in enhancing their knowledge. Enjoyment of any form of online learning could be due to the flexibility it provides to the students. Moreover, enjoyment of learning can have a direct effect on the enhancement of knowledge. This fact was also pointed out by Ma et al., in their study where most of the students found the blended learning method enjoyable and helped them increase their knowledge and skills [14].

Time management among medical graduates is crucial as it can have a direct impact on learning [15]. Optimal time management is essential not just for achieving high grades but also to attain quality educational outcomes [16]. Various studies have pointed out that the exponential increase in knowledge within a defined time limit has been identified as a challenge for medical graduates. For this reason, medical institutes had to adopt newer teaching learning methodologies [17]. In this context, any  major change in the mode of learning warrants an inquiry regarding time management. In our study, the students both boys and girls were of the opinion that their time management was better with the asynchronous method as compared to the synchronous method (*P* < 0.001) (Tables 1 and 2). This was probably due to the fact that in asynchronous learning, the reading materials were given before-hand and discussion was done later. This helped the students to plan their schedule and work according to their convenience rather than attend live lectures at a particular time as required  in synchronous learning. Having the freedom and flexibility to plan their studies could also be the reason why the student found asynchronous learning easier and more convenient as compared to synchronous learning. Although this was opined by both boys and girls, the response of female students was statistically significant (*P* = 0.001). Similar to this, studies done on students’ perspectives towards asynchronous learning showed that asynchronous learning gave them flexibility in time and place as well [18, 19].

Effective learning depends upon good interaction. Asking questions, giving explanations, providing timely feedback, and group activities among students are some crucial communication that enhance learning [20]. Although face to face elements interaction is an integral part of traditional classroom teaching, various systems are being adopted to improve interaction in online learning enviroments. In fact, the quality of interaction between a learner and an online facilitator has been considered as one of the vital components which determines the efficacy of online learning [21]. Hence, we enquired about the students’ view on interaction during both asynchronous and synchronous learning. Our study showed that the students, girls in particular (*P* = 0.03) found the synchronous method to be more interactive than the asynchronous method. This was because the synchronous method involved live online lectures in real time just like classroom teaching, sans face to face interaction. This enabled the teacher and students to ask questions during the ongoing lectures rather than post questions through the discussion board of the LMS. There are some limitations to our study. This study was conducted on the final year student of one medical college. Moreover, it was done only regarding the surgery lectures. Therefore, similar studies from other departments and colleges would be necessary to generalize the findings.

## Conclusion

The study participants found both synchronous and asynchronous learning equally good regarding effectiveness and comfort, and both modes were reported to be equally enjoyable. Students perceived asynchronous learning more advantageous as regards time-management. On the other hand, synchronous learning was reported to be better at facilitating interactiveness during the learning session. In the light of these advantages/limitations of the studied modes of instruction as analysed by this study, an integrated approach comprising of both synchronous and asynchronous elements may be adopted to plan any module/course depending upon the contents of the topic and the learning outcomes.

## Data Availability

The datasets and/or analyzed during the current study available from the corresponding author on reasonable request due to privacy concerns.
